# Development of a bioassay to detect T-cell-activating impurities for T-cell-dependent bispecific antibodies

**DOI:** 10.1038/s41598-019-40689-1

**Published:** 2019-03-07

**Authors:** Ho Young Lee, Edward Contreras, Ames C. Register, Qiang Wu, Kathleen Abadie, Khristofer Garcia, Pin Yee Wong, Guoying Jiang

**Affiliations:** Biological Technologies, Department of Analytical Development and Quality Control, Genentech-a Member of the Roche Group, South San Francisco, California, 94080 USA

## Abstract

T-cell-dependent bispecific antibodies (TDBs) are promising cancer immunotherapies that recruit a patient’s T cells to kill cancer cells. There are increasing numbers of TBDs in clinical trials, demonstrating their widely recognized therapeutic potential. Due to the fact that TDBs engage and activate T cells via an anti-CD3 (aCD3) arm, aCD3 homodimer (aCD3 HD) and high-molecular-weight species (HMWS) are product-related impurities that pose a potential safety risk by triggering off-target T-cell activation through bivalent engagement and dimerization of T-cell receptors (TCRs). To monitor and control the level of unspecific T-cell activation, we developed a sensitive and quantitative T-cell-activation assay, which can detect aCD3 HD in TDB drug product by exploiting its ability to activate T cells in the absence of target cells. This assay provides *in-vivo*-relevant off-target T-cell-activation readout. Furthermore, we have demonstrated that this assay can serve as a platform assay for detecting T-cell-activating impurities across a broad spectrum of aCD3 bispecific molecules. It therefore has the potential to significantly benefit many T-cell-recruiting bispecific programs.

## Introduction

Bispecific antibodies are large-molecule therapeutics that target two different antigens simultaneously, maximizing therapeutic efficacy either synergistically or additionally. Currently, more than 30 bispecific antibodies are under early clinical development^1,2^, spanning a diverse range of mechanisms of action: recruiting T cells^3–6^, blocking signaling pathways simultaneously, accommodating or inhibiting association of two factors, and independently binding to ligands or receptors^7–11^. Furthermore, bispecific antibodies have been developed in a variety of structural formats, including IgGs 1, 2, and 4; Fc fusions; multivalent monoclonal antibodies (mAbs); F(ab′)2 fragments; and nanobodies, among others^12–24^. Combined with their enhanced therapeutic efficacy, the structural and functional diversity that can be designed into bispecific antibodies make them a versatile and promising avenue for drug development.

In contrast to conventional mAbs, bispecific antibodies are heterodimers, which presents unique purification challenges. Manufacturing processes inevitably yield varying amounts of homodimers of each monovalent arm, in addition to unassembled half-mAb^25–29^. Homodimer impurities often possess physical properties (size, charge, binding affinity, etc) similar to those of the heterodimeric bispecific product, making them difficult to identify and separate by conventional means. Developing efficient processes for removal of homodimers from final products and designing an appropriate control system strategy are critical for successful development of bispecific molecule therapeutics^28,30–32^.

It is particularly important to remove and quantitate homodimer impurities in light of their potential safety concerns. These concerns are especially clear in T-cell-dependent bispecific antibodies (TDBs)—a promising emerging class of cancer immunotherapy. TDBs (also known as T-cell-recruiting bispecific antibodies) harness a patient’s own immune system to kill tumor cells^4,33–35^. When a TDB binds to its target antigen on a tumor cell and to a T-cell receptor (TCR) on a T cell, the TDB triggers T-cell activation and subsequent killing of the target cell. The activity of TDBs is dependent on the engagement of target cells, and no cell-killing activity is observed in the absence of target cells^4,36–38^. TDBs engage T cells via an anti-CD3 (aCD3) arm that binds the TCR complex on the surface of T cells. For these bispecifics, aCD3 homodimer (aCD3 HD) poses a significant safety risk due to its ability to activate T cells in the absence of target cells by TCR dimerization. This off-target (also known as target-cell-independent) T-cell activation can induce cytokine secretion, triggering an undesired immune response in patients. Off-target T-cell activation by aCD3 HD is an undesired activity and distinct from the T-cell activation by TDB, which eventually kills the target tumor cells. Therefore, it is important to control the level of aCD3 HD in TDB drug products, and sensitive assays are needed to enable its quantitation. In addition to quantitating aCD3 HD, cell-based assays that can measure off-target T-cell activation would be useful for characterizing the biological effects of aCD3 HD and other T-cell-activating impurities that may be present in TDB drug products.

To measure the level of aCD3 HD and also assess its therapeutic risk, we have developed a novel cell-based T-cell-activation assay, which is responsive to off-target T-cell activation by aCD3 HD. While developing the assay we found that other impurities can also contribute to off-target T-cell activation, and we used our assay to characterize their relative impacts. We demonstrate that our assay is accurate and sensitive while providing *in-vivo*-relevant quantitation of T-cell activation by impurities. This represents the first T-cell-activation assay for detection of T-cell-activating impurities, including aCD3 HD, and provides valuable insight into the overall contribution of impurities to off-target T-cell activation.

## Results and Discussion

### Developing T-cell-activation assay for aCD3 homodimer quantitation

In nature, when T cells are activated they secrete cytokines, which are instrumental in coordinating the immune system’s response to infection. Expression of many of these cytokines occurs via the well-characterized nuclear factor kappa-light-chain-enhancer of activated B cells (NFkB) pathway. Under normal conditions, the NFkB transcription factor is prevented from binding the NFkB promoter through its interaction with IkBa, and gene expression is repressed. In response to T-cell-activating signals, IkBa is ubiquitinylated and degraded, freeing NFkB to bind its promoter and facilitate expression of genes encoding cytokines and other immune-responsive factors^39,40^. Therefore, expression of genes under the control of the NFkB promoter can serve as a readout of T-cell activation upstream of target-cell killing.

**Figure 1:**
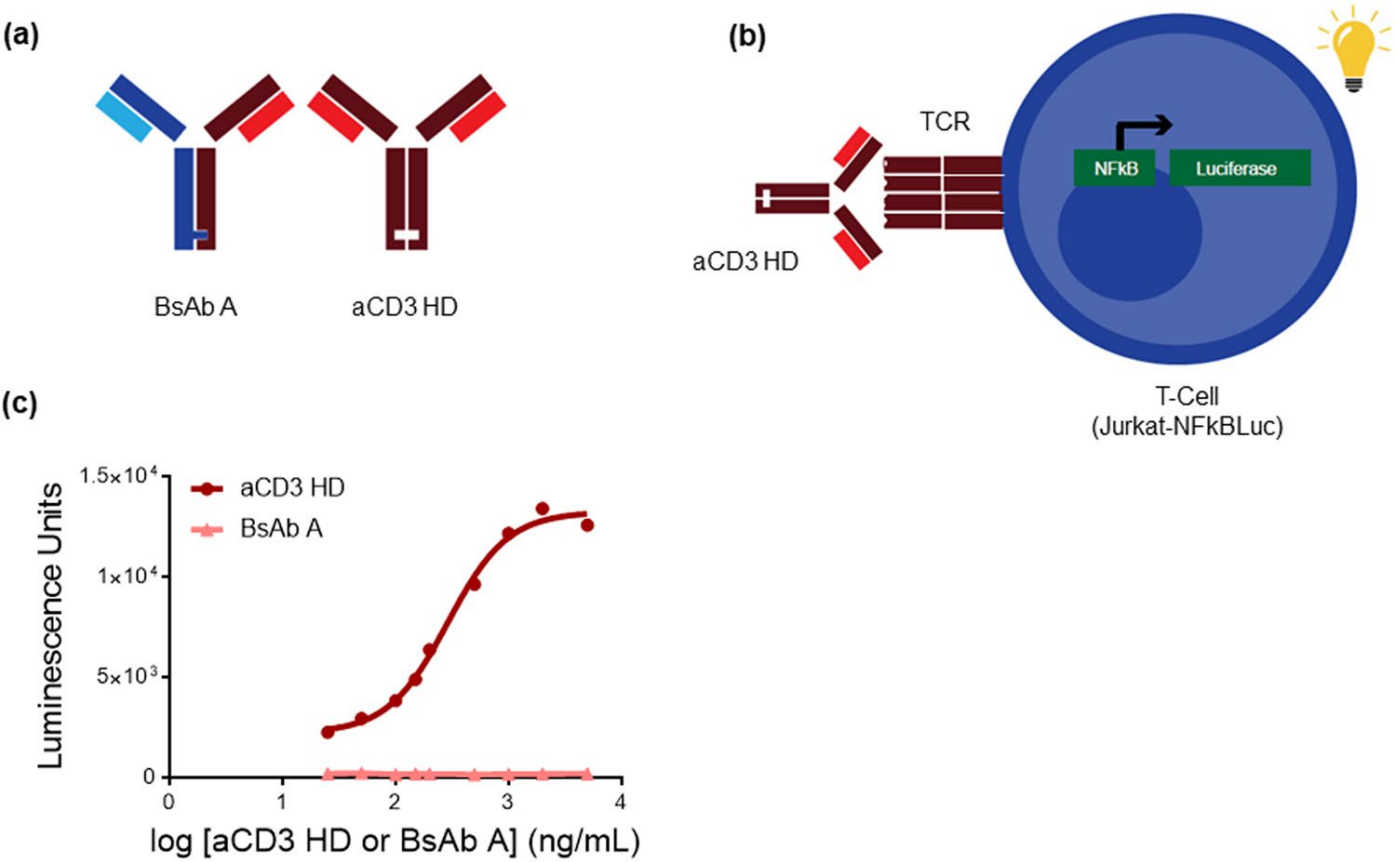
aCD3 homodimer can activate T-cells in the absence of target cells. **(a)** Structure of BsAb A and aCD3 homodimer (HD). **(b)** Assay schematic of T-cell-activation assay, which utilizes Jurkat reporter-gene cell line engineered to express luciferase upon T-cell activation. **(c)** Standard dose-response curve of aCD3 HD (circle) and BsAb A (triangle) in T-cell-activation assay.

To develop an assay for aCD3 HD, we used a Jurkat T-cell line, which expresses the Luciferase A gene under NFkB promoter (see Materials and Methods). When Jurkat cells are activated by aCD3 HD binding to and dimerizing TCR, luciferase is expressed in a dose-responsive manner to aCD3 HD (Fig. 1b).

To capture off-target T-cell activation, only effector T cells are used for the assay (no target cells are present). The monovalent interaction of the TDB molecule to TCR on the effector cell is not sufficient to activate T cells without target-cell engagement. In contrast, the bivalent aCD3 HD interaction effectively dimerizes TCRs, resulting in T-cell activation (Fig. 1a,c). Thus, the assay is able to distinguish aCD3 HD from the bispecific molecule, BsAb A—the major species in the final product.

### Reporter gene readout correlates to CD-marker expression in the T-cell-activation assay

**Figure 2:**
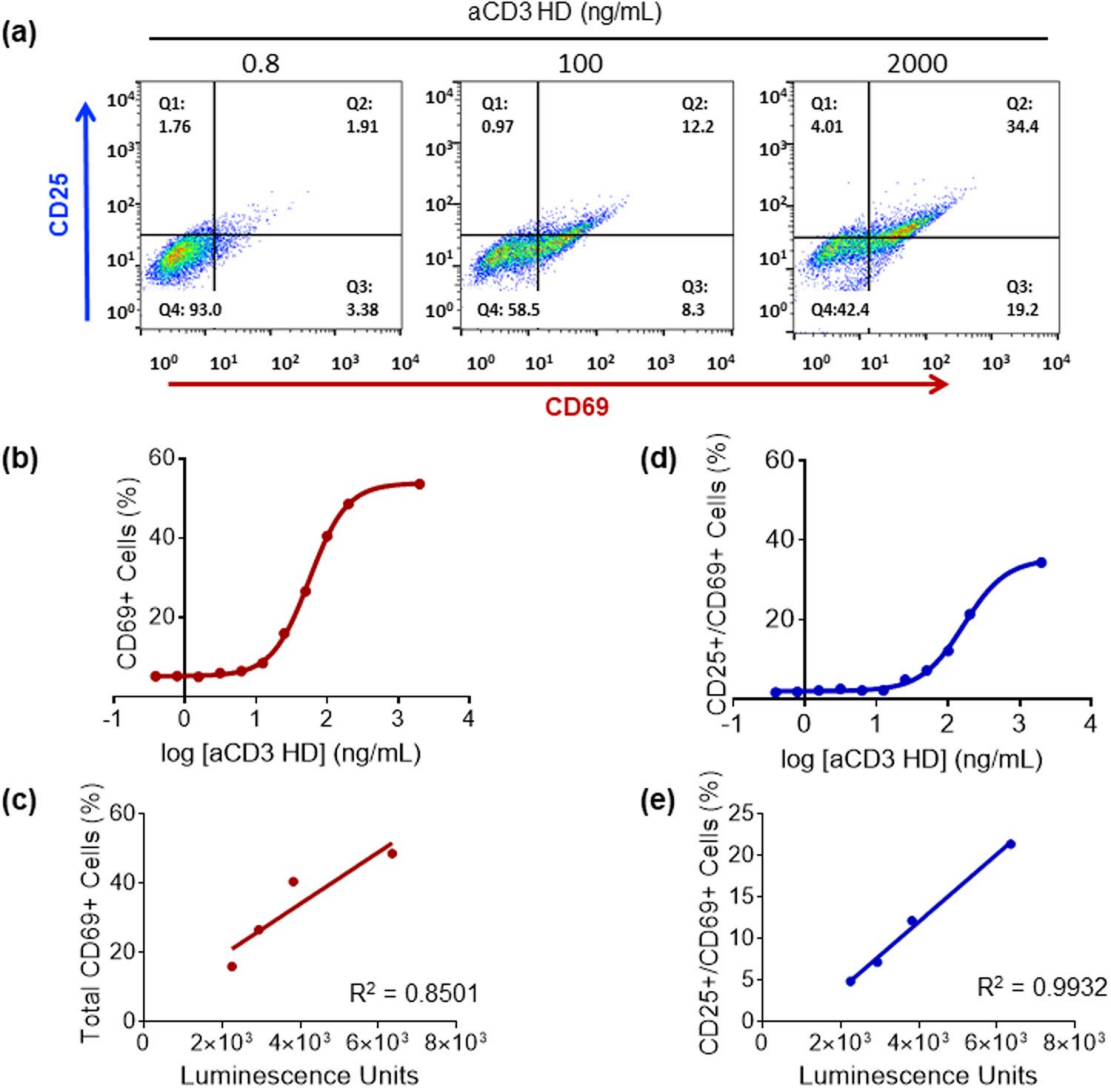
Expression of CD markers shows correlation with luminescence signal from T-cell activation. **(a)** Flow-cytometric analysis of expression of CD markers CD25 and CD69 on T cells. **(b)** CD69-expressing cells increase with increasing amounts of aCD3 HD. **(c)** The correlation between CD69+ cells and luciferase expression in the T-cell-activation assay. **(d)** CD69- and CD25-expressing cells increase with increasing amounts of aCD3 HD. **(e)** The correlation between CD69+ CD25+ cells and luciferase expression in the T-cell-activation assay.

As our assay measures T-cell activation indirectly through a surrogate signal—luciferase expression—we wanted to confirm that the assay readout is a true indicator of T-cell activation. When a T-cell is activated it expresses characteristic CD markers: CD25 and CD69. Using fluorescence-activated cell sorting (FACS), we measured the expression levels of CD25 and CD69 with increasing dose of aCD3 HD (Fig. 2) and compared the results to luciferase expression in the T-cell-activation assay. Luciferase expression and CD marker expression are highly correlated (Fig. 2b,d), confirming that the luciferase signal from this assay is a true indication of T-cell activation by aCD3 HD binding. CD25 expression (data not shown) was rather weak compared to CD69 expression in our assay, as CD25 expression takes longer (>1 day) than the given assay incubation time while CD69 expresses in several hours upon T-cell activation.

### TDB interferes with aCD3 homodimer-mediated T-cell activation

**Figure 3:**
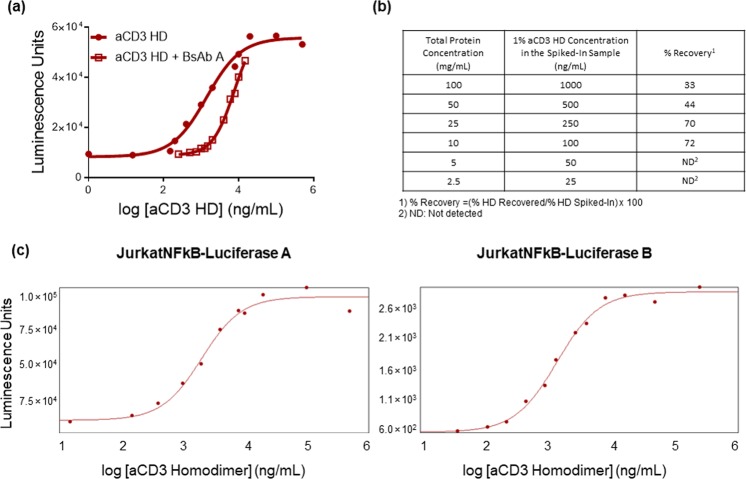
BsAb interferes with aCD3 HD quantitation in the T-cell-activation assay. **(a)** Standard curves of aCD3 HD and aCD3 HD spiked into BsAb A. **(b)** Table listing percent recovery of spiked-in aCD3 HD with varying total protein concentration; recovery is limited by assay sensitivity **(c)**. Dose-response curves of Luciferase A cell line and Luciferase B cell line: Luciferase B cell line demonstrates higher sensitivity than Luciferase A cell line.

During initial assay development, we used enriched, purified aCD3 HD and observed a strong dose response of T-cell activation to aCD3 HD. However, our product contains mostly TDB (BsAb A) and relatively low amounts of aCD3 HD after purification, so we tested the assay with more product-like samples, containing mostly BsAb A, with low, known quantities of aCD3 HD. Interestingly, we found that the TDB competes with aCD3 HD for TCR binding, showing concentration-dependent interference in the T-cell activation signal (Fig. 3a). The interference by BsAb A results in lower quantitation of homodimer than what is known to be present (i.e., lower recovery of aCD3 HD). The interference is more severe when the total protein concentration is higher and/or aCD3 HD content is relatively lower—conditions in which there is more BsAb A to compete with aCD3 HD for access to TCRs. Unfortunately, because TDB interference varies with aCD3 HD concentration, it is not possible to apply a single correction factor to account for TDB interference. To overcome interference from BsAb A, we diluted the samples and measured the percent recovery of spiked-in homodimer of each dilution. Recovery of spiked-in aCD3 HD increased with dilution factor (Fig. 3b), but the assay detection limit was reached before full recovery was achieved. This suggests the possibility of overcoming BsAb A interference to achieve accurate aCD3 HD quantitation by decreasing total protein concentration; however, a more sensitive assay system would be required, capable of detecting low concentrations of aCD3 HD in majority-BsAb A samples.

### Improved assay sensitivity by using an alternative cell line with secreted Luciferase B

In order to increase assay sensitivity, we evaluated an alternative reporter gene cell line, which expresses Luciferase B with a secretion tag when activated. Compared to the previously used Jurkat cell line expressing Luciferase A, the secreted Luciferase B cell line greatly increased the sensitivity of the assay, lowering the limit of detection (LOD) up to 10-fold (measured by comparing the EC10 [LOD] of each dose-response curve) (Fig. 3c). This could be from the higher sensitivity of the Luciferase B compared to Luciferase A; Luciferase B is a different luciferase protein from a different organism and uses a highly sensitive substrate compared to the substrate of Luciferase A^41^.

Also, the secretion tag attached to Luciferase B allows for higher levels of Luciferase B accumulation than endogenous Luciferase A, which may be prone to degradation.

For accurate aCD3 quantitation with minimum interference from BsAb A, we designed assay standards with different levels of aCD3 spiked into BsAb A drug product but with total protein concentration fixed at the same concentration as the samples for testing. By comparing the signal of aCD3 homodimer from the sample to the standard curve, the level of aCD3 homodimer in samples can be quantitated.

**Figure 4:**
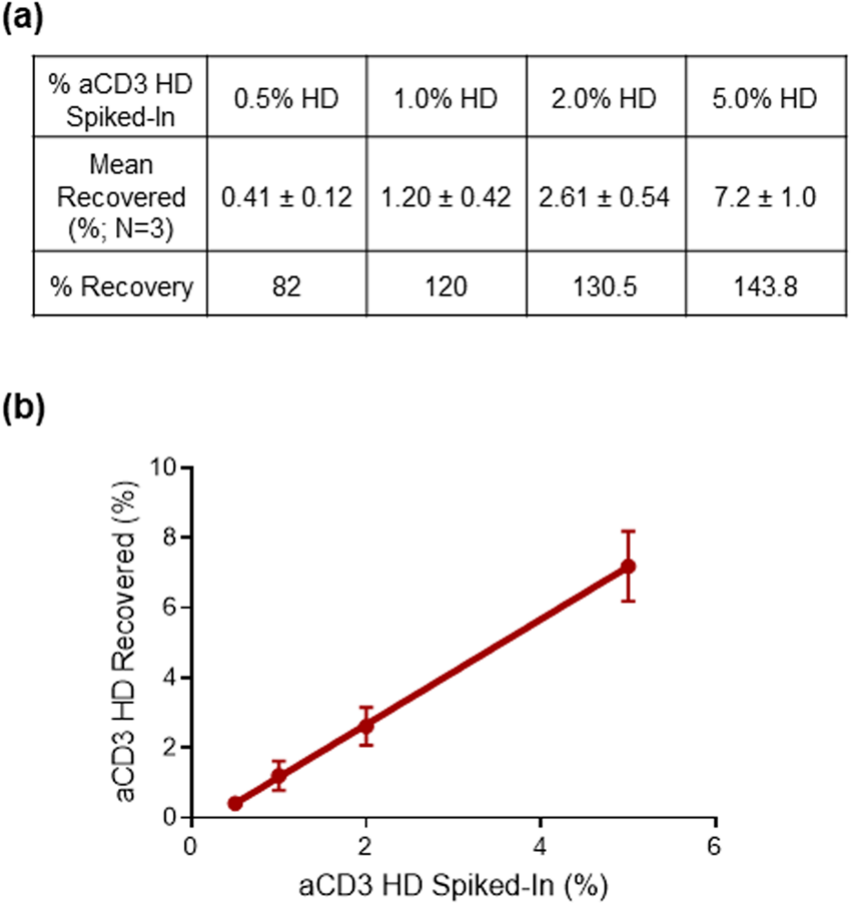
aCD3 HD quantitation and recovery using the aCD3 HD spiked into BsAb A samples. **(a)** aCD3 HD qualification results table showing percent recovery of aCD3 HD spike-in samples. **(b)** Correlation between percent recovered and percent spiked in.

Assay qualification results, in which a known amount of aCD3 HD was spiked into BsAb A samples and incubated with the Luciferase B cell line, demonstrated that the assay can detect aCD3 HD as low as 0.5% of total protein, with improved accuracy and precision compared to that observed with the Luciferase A cell line. Linearity was also improved, with an R^2^ of 0.999 in the relevant aCD3 HD range between 0.5% and 5%. (Fig. 4).

### Trace aCD3 HD impurity in BsAb A contributes to T-cell activation, leading to over-recovery of aCD3 HD in spike-in experiments

**Figure 5:**
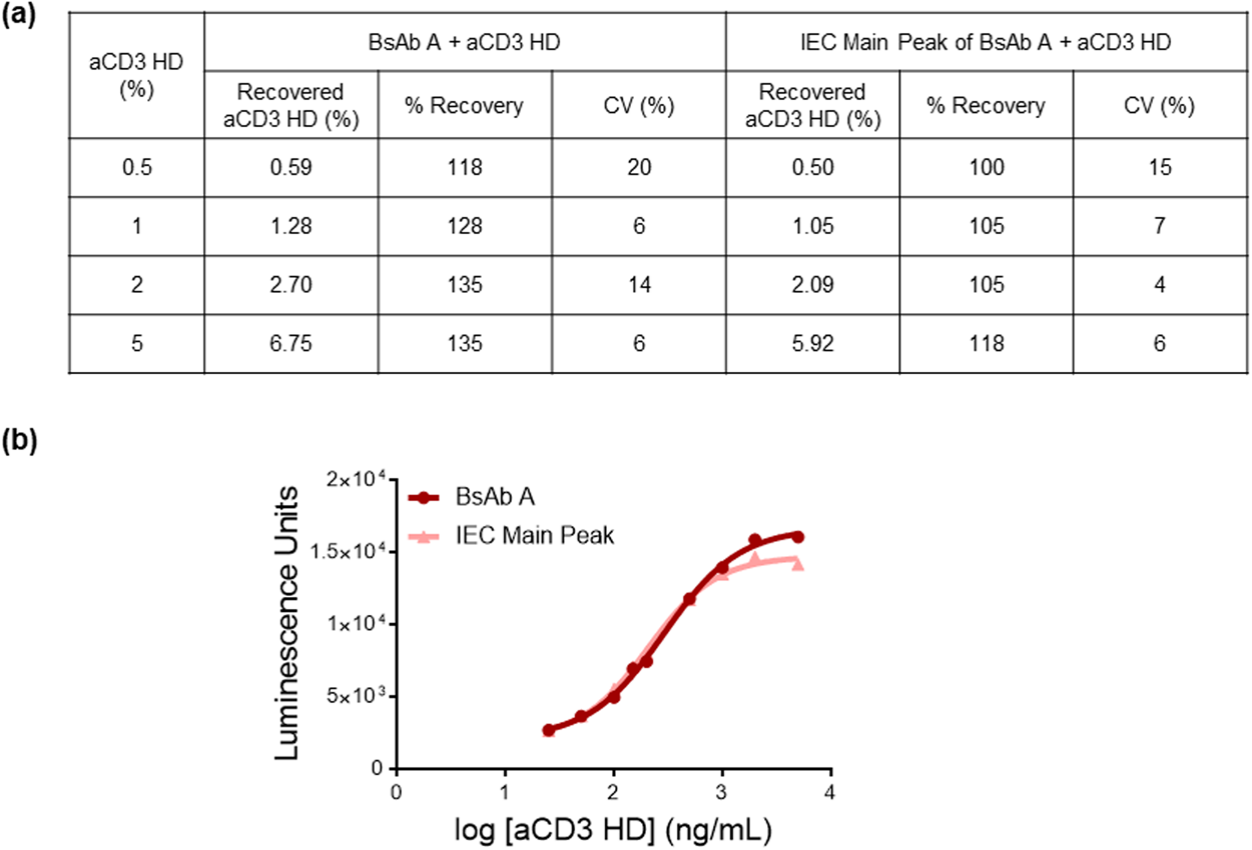
BsAb A with the highest purity provides accurate quantitation of aCD3 HD present. **(a)** aCD3 HD recovery (%) when spiked into BsAb A drug product vs. into the isolated IEC main peak of BsAb A. **(b)** Standard curves of aCD3 HD spiked into BsAb A (circle) and into the IEC main peak of BsAb A (triangle).

After performing a number of aCD3 HD spike-in experiments, we noticed that percent recoveries were greater than 100% at all levels of spiked-in aCD3 HD (Fig. 5a). One possible explanation for the observed over-recovery is that the BsAb A reference material into which the purified aCD3 HD is added contains small amounts of aCD3 HD impurity. This aCD3 HD impurity could be contributing to T-cell activation and increasing the calculated HD recovery. To test this hypothesis, we performed identical spike-in experiments, but instead of adding aCD3 HD to BsAb A reference material, HD was spiked into BsAb A isolated from the main peak of an ion-exchange chromatography (IEC) separation of BsAb A. This IEC main peak BsAb A is of higher purity, with no aCD3 HD detected by mass spectrometry (data not shown). Percent recoveries of aCD3 HD spiked into the BsAb A from isolated IEC main peak are much closer to 100% (Fig. 5).

### Contribution of other T-cell-activating impurities to off-target T-cell activation

Because our assay detects T-cell activation, impurities other than aCD3 HD that are present in TDB drug product can potentially contribute to assay readout. To address this question, we characterized three key impurities that may potentially contribute to T-cell activation—endotoxin, host cell proteins (HCP), and high-molecular-weight species (HMWS)—elucidating their contributions to T-cell activation both alone and in combination with aCD3 HD.

**Figure 6:**
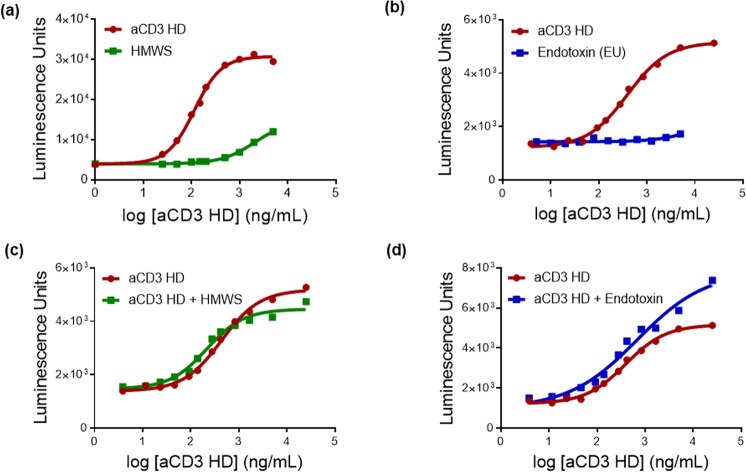
T-cell-activating impurities other than aCD3 HD contribute to T-cell-activation assay. **(a)** HMWS (square) vs. aCD3 HD (circle) in the T-cell-activation assay. **(b)** Endotoxin (square) vs. aCD3 HD (circle) in T-cell-activation assay. Highest concentration of endotoxin is 50 endotoxin units (EU); each subsequent point represents a two-fold dilution from the previous point. **(c)** aCD3 HD+ HMWS (1:1 co-mixture) (square) vs. aCD3 HD (circle); graphs plotted based on the amount of aCD3 HD present. **(d)** aCD3 HD+ Endotoxin (10 EU) (square) vs. aCD3 HD (circle); graphs plotted based on the amount of aCD3 HD present.

HMWS activate T cells in a target-cell-independent manner analogous to aCD3 HD. However, the level of T-cell activation is lower compared to aCD3 HD (Fig. 6a). The avidity of HMWS with multiple aCD3 armsallows cross-linking of TCRs, but they may not do so as efficiently as aCD3 HD, perhaps due to conformational differences.

Endotoxin and HCP (HCP data not shown) did not produce any T-cell activation in the absence of target cells, suggesting that they are not relevant T-cell-activating impurities by themselves (Fig. 6b).

However, when co-present with aCD3 HD, each of the three impurities triggered a higher level of T-cell activation compared both to their individual signal and to aCD3 HD alone (Fig. 6c,d). This result suggests that there is synergistic target-cell-independent T-cell activation when impurities interact with T cells simultaneously. However, the relative contribution of each impurity when co-present is not clearly understood.

These results demonstrate that impurities such as HMWS and endotoxin can impact aCD3 HD quantitation. Thus, our T-cell-activation assay is not aCD3 HD specific, but rather a “catch-all” method for detecting all T-cell-activating impurities present in TDB drug substance or product. As the levels of the non-aCD3-HD impurities are robustly controlled at low levels by specific analytical assays (size-exclusion chromatography [SEC], limulus amoebocyte lysate test, and HCP ELISA), the level of T-cell activation by multiple T-cell-activating impurities are reported as the relative percent aCD3 HD.

As a “catch-all” assay, this assay provides conservative and orthogonal information on the combined effect of multiple T-cell-activating impurities on off-target T-cell activation, which might not be assessable by individual assays of each impurity.

### Platform approach for multiple T-cell-dependent bispecifics

**Figure 7:**
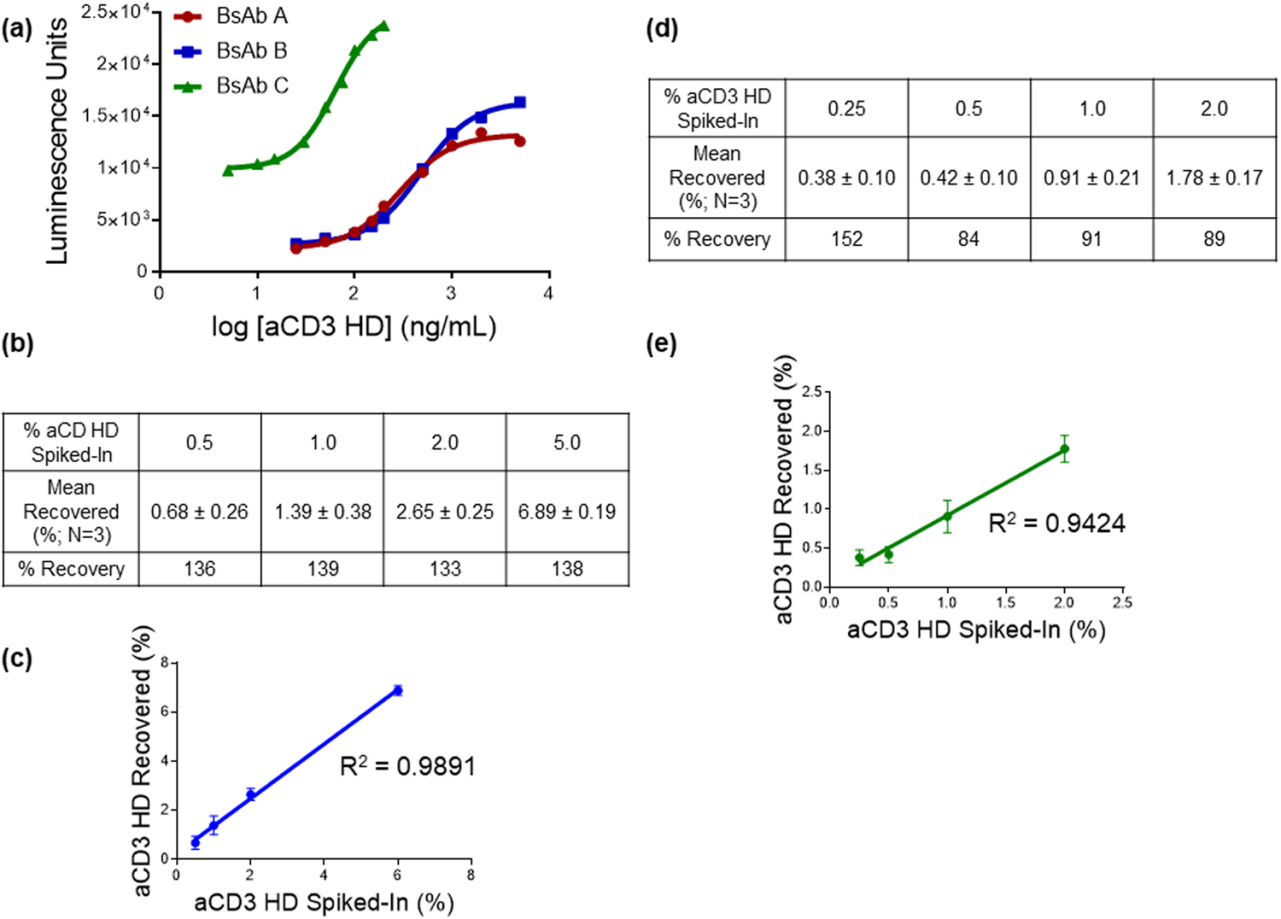
Platform use of the T-cell-activation assay for different T-cell-dependent bispecific molecules. **(a)** Dose-response standard curves of the aCD3 HD corresponding to BsAb A (red circle), BsAb B (blue square), and BsAb C (green triangle). BsAb A and BsAb B share the same aCD3 arm. BsAb C has a different aCD3 arm. For BsAb C T-cell-activation assay, the assay incubation time was increased for the best assay result. **(b)** BsAb B qualification results table showing percent recovery of spiked-in aCD3 HD. **(c)** BsAb B correlation between percent recovered and percent spiked-in. **(d)** BsAb C qualification results table showing percent recovery of spiked-in aCD3 HD. **(e)** BsAb C correlation between percent recovered and percent spiked-in.

Given the fact that many TDBs under clinical development bind TCR via an aCD3 arm, this assay has the potential to serve as a platform assay for detection and quantitation of aCD3 HD impurity across many TDBs currently in development. To evaluate our assay’s utility as a platform assay, we applied our method to two different bispecific molecules: BsAb B, a TDB possessing the same aCD3 arm as BsAb A (the TDB used in assay development), and BsAb C, a TDB possessing a different aCD3 arm with higher affinity compared to BsAb A. As BsAb A and BsAb B have identical aCD3 HD impurity in their drug products, a simple assessment was carried out to demonstrate that the assay can be applied to BsAb B without changing the experimental conditions originally developed for BsAb A (Fig. 7a,b). Due to its distinct aCD3 arm, applying the assay to quantitation of aCD3 HD in BsAb C drug product required minor optimization and adjusting the concentration range to cover the full dose-response curve (Fig. 7a). Overall, we have shown that the assay can be applied to aCD3 HD detection for BsAb C, demonstrating that our T-cell-activation assay is broadly applicable as a platform assay for multiple aCD3-engaging TDBs despite differences in the aCD3 arm (Fig. 7c). There have been efforts to reduce mispairing or homodimer formation, for example by implementing a knob-into-hole-format, but it is known that the knob-and-hole format could still form homodimers^42^.

## Conclusion

aCD3 HD is a unique product-related impurity for CD3-targeting bispecific antibodies. In contrast to other product-related impurities, it is structurally highly similar to the product bispecific molecules, presenting technical challenges in developing analytical assays and posing a potential safety risk for immunogenicity via off-target T-cell activation. Furthermore, it is a significant challenge to develop an assay sensitive enough to detect the low levels of aCD3 HD and other T-cell-activating impurities present in TDB products.

This work describes the development of a T-cell-activation assay capable of detecting and quantitating low levels of T-cell-activating impurities in TDB product by exploiting the ability of aCD3 HD to activate T cells in the absence of target cells by dimerizing TCR on the surface of T cells. This assay is a “catch-all” T-cell-activating impurity assay – quantitating the total amount of T-cell-activating impurities in terms of percent aCD3 HD. As this assay is designed based on the biological impact of the aCD3 HD (off-target T-cell activation), it provides important information regarding the *effective* level of T-cell-activating impurities. This information is more relevant to the *in-vivo* safety concern of target-independent T-cell activation than is the absolute quantitation of aCD3 HD or HMWS content by physicochemical methods and provides key functional information that is not accessible by those methods. Our finding that the presence of endotoxin or HMWS can have synergistic, difficult-to-predict effects on off-target T-cell activation highlights the need for assays that have biologically relevant readouts when assessing TDB product safety. Furthermore, the use of an engineered reporter-gene cell line to measure T-cell activation makes the assay faster and easier to perform as well as more reproducible than analogous assays based on peripheral blood mononuclear cells.

Finally, the fact that many TDBs engage T cells via an aCD3 arm motivated us to demonstrate that this assay can be used to quantitate T-cell-activating impurities in multiple CD3-targeting bispecifics currently under development with relatively minor, straight-forward optimization. This demonstrates that our T-cell-activation assay has the potential to serve as a platform assay for a broad spectrum of aCD3 bispecific molecules and could provide valuable information about potential safety concerns for other TDB programs.

## Materials and Methods

### BsAb A, BsAb B, BsAb C

BsAb A, BsAb B, and BsAb C were produced as full-length human IgG1 in a knob-into-hole format, as previously described^43^.

### JurkatNFkB-Luciferase A cell line

NFkB transcriptional response element was subcloned through standard molecular biology methods into a lentiviral expression vector upstream of a minimal CMV promotor and Luciferase A gene. Jurkat cells were subjected to lentiviral transfection and individual clones were isolated and screened for inducible luciferase expression. Cells were cultured in RPMI 1640 medium containing 10% heat-inactivated fetal bovine serum (HI FBS), 1x Pen-Strep, 1x GlutaMAX^TM^, and 1 µg/mL puromycin.

### JurkatNFkB-Luciferase B cell line

A single clone was isolated from the Jurkat cell line with stable integration of an inducible reporter construct, Luciferase B under NFkB. Cells were cultured in RPMI 1640 medium containing 10% HI FBS, 0.5x Pen-Strep, 1x GlutaMAX^TM^, 100 µg/mL zeocin, and 10 µg/mL blasticidin.

### aCD3 homodimer

Protein-A affinity chromatography pool from the harvested cell culture fluid of the aCD3 half antibody was used to isolate the aCD3 homodimer. The aCD3 homodimer present in the affinity pool was purified by a method that includes purification by POROS cation exchange chromatography followed by ultrafiltration/diafiltration into the final desired formulation.

### HMWS, endotoxin, and HCP

HMWS was isolated from the final product or the stressed sample by collecting fractions using SEC. Endotoxin was purchased from Charles River (Cat No. E120, Control Standard Endotoxin). HCP was prepared from Chinese hamster ovary cell culture fluid at the 400 L scale. The resulting harvested cell culture fluid was concentrated ~10-fold, diafiltered against phosphate-buffered saline (PBS) for 6 volumes on a 30,000 Da molecular-weight-cutoff membrane, and aliquoted.

### T-cell-activation assay procedure

Sample dilutions of TDB were prepared in an assay medium consisting of RPMI 1640 (no phenol red) supplemented with 10% HI FBS. Dilutions for the standard curve were prepared in the assay medium by spiking increasing amounts of aCD3 HD into TDB drug product. In the same 96-well tissue culture plate, both sample and standard dilutions were incubated with JurkatNFkB-Luciferase B cells in assay medium for 18 hours in a 37 °C incubator with 5% CO_2_. After incubation, plates were equilibrated to room temperature for 15 minutes with shaking. 50 µL from each well was transferred to a new plate, and 50 µL of QUANTI-Luc^TM^ reagent was added to each well. Plates were shaken for 3–5 minutes at room temperature before measuring luminescence (in relative luminescence units [RLU]) using a suitable plate reader. The results for the standard curve were plotted as RLU versus percent aCD3 HD using Softmax® Pro software.

For accurate aCD3 HD quantitation with minimum interference from BsAb A, the assay standard dilutions contain different levels of spiked-in aCD3 HD in BsAb A drug product with fixed total protein concentration of 10 µg/mL. The sample was diluted to 10 µg/mL or lower concentration. The percentage of T-cell-activating impurity in each sample was interpolated from the standard curve by comparing the signal of aCD3 HD from the sample to the standard curve. The level of aCD3 HD in samples can then be quantitated and expressed as percent aCD3 HD. Samples below the assay LOD (EC10) were reported as <0.5% aCD3 HD.

### CD25 and CD69 expression level by FACS

Dilutions of aCD3 HD were prepared in assay medium consisting of RPMI 1640 supplemented with 10% HI FBS. 0.8 mL of each dilution was transferred to individual wells of 12-well tissue culture plates, and then pre-incubated at 37 °C for one hour. After pre-incubation, 0.8 mL of JurkatNFkB-Luciferase B cells at 2 × 10^6^ cells/mL were added to each well. Plates were incubated overnight in a 37 °C incubator with 5% CO_2_. Cells were harvested by centrifugation (300 × g, 5 minutes) and washed twice with cold PBS lacking Ca^2+^ and Mg^2+^ before being resuspended in cold PBS at a concentration of about 2 × 10^6^ cells/mL and labeled with PE-antiCD25 and FITC-antiCD69 (5 ng/mL) for 30 minutes on ice. Labeled cells were analyzed by FACS using a FACScalibur^TM^ instrument, and data were processed using FlowJo® software.
